# Molecular profiles of high‐grade and low‐grade pseudomyxoma peritonei

**DOI:** 10.1002/cam4.542

**Published:** 2015-10-16

**Authors:** Rei Noguchi, Hideaki Yano, Yoshimasa Gohda, Ryuichiro Suda, Toru Igari, Yasunori Ohta, Naohide Yamashita, Kiyoshi Yamaguchi, Yumi Terakado, Tsuneo Ikenoue, Yoichi Furukawa

**Affiliations:** ^1^ Division of Clinical Genome ResearchThe Institute of Medical ScienceThe University of TokyoTokyoJapan; ^2^ Department of SurgeryNational Center for Global Health and MedicineTokyoJapan; ^3^ Pathology Division of Clinical LaboratoryNational Center for Global Health and MedicineTokyoJapan; ^4^ Department of Pathology, Research HospitalThe Institute of Medical ScienceThe University of TokyoTokyoJapan; ^5^ Department of Advanced Medical ScienceResearch HospitalThe Institute of Medical ScienceThe University of TokyoTokyoJapan

**Keywords:** DPAM, mutation, PMCA, pseudomyxoma peritonei, TP53

## Abstract

Pseudomyxoma peritonei (PMP) is a rare disease exhibiting a distinct clinical feature caused by cancerous cells that produce mucinous fluid in the abdominal cavity. PMPs originate most frequently from the appendix and less frequently from the ovary. This disease can range from benign to malignant, and histologically, PMP is classified into two types: disseminated peritoneal adenomucinosis (DPAM) representing the milder phenotype, and peritoneal mucinous adenocarcinomas (PMCA) representing the aggressive phenotype. Although histological classification is clinically useful, the pathogenesis of PMP remains largely unknown. To elucidate the molecular mechanisms underlying PMP, we analyzed 18 PMP tumors comprising 10 DPAMs and 8 PMCAs. DNA was extracted from tumor and matched non‐tumorous tissues, and was sequenced using Ion AmpliSeq Cancer Panel containing 50 cancer‐related genes. Analysis of the data identified a total of 35 somatic mutations in 10 genes, and all mutations were judged as pathological mutations. Mutations were frequently identified in *KRAS* (14/18) and *GNAS* (8/18). Interestingly, *TP53* mutations were found in three of the eight PMCAs, but not in the DPAMs. *PIK3CA* and *AKT1* mutations were also identified in two PMCAs, but not in the DPAMs. These results suggested that *KRAS* and/or *GNAS* mutations are common genetic features of PMP, and that mutations in *TP53* and/or genes related to the PI3K‐AKT pathway may render malignant properties to PMP. These findings may be useful for the understanding of tumor characteristics, and facilitate the development of therapeutic strategies.

## Introduction

Pseudomyxoma peritonei (PMP) is a disease exhibiting clinical manifestations such as extended abdomen, abdominal discomfort, obstruction in digestive tract, and nutritional compromise due to accumulated gelatinous ascites and/or disseminated lesions of neoplastic mucin‐secreting cells in the peritoneal cavity. Primary tumor cells of PMP develop most frequently in the appendix and occasionally in other organs including the ovary, colorectum, gallbladder, stomach, pancreas, fallopian tube, urachus, lung, and breast 1. It is a rare condition with an estimated incidence of 1–2 per million per year 2. Based on their architectural complexity and degree of cytologic atypia, the tumors are histologically divided into two groups: 3 disseminated peritoneal adenomucinosis tumors (DPAM) showing low‐grade cytologic atypia (nucleomegaly, nuclear stratification, rate mitotic figures, and single cell necrosis) and minimal architectural complexity (villiform, flat epithelial proliferation, and small papillary excrescences), and peritoneal mucinous adenocarcinomas tumors (PMCA) demonstrating any of the following: destructive invasion of the organ's wall, high‐grade cytologic atypia (extensive full‐thickness nuclear stratification, vesicular nuclei, marked nuclear membrane irregularities, prominent nucleoi, and brisk mitotic activity), or complex epithelial proliferation (complex papillary fronds and cribriform glandular spaces). The histological classification is reportedly associated with prognosis of the patients with PMP; a median 5‐year survival of 62.5% in DPAMs and 37.7% in PMCAs 4.

Several molecular studies have disclosed profiles of PMP including frequent mutations in *KRAS* and *GNAS*, decreased expression of E‐cadherin, and amplification of *MCL1* and *JUN*. However, the precise molecular mechanism(s) underlying the development and progression of PMP and genetic factors associated with the response of their treatment remain to be resolved.

In this study, we have analyzed a total of 18 Japanese PMPs comprising 10 DPAMs and 8 PMCAs using multiplex‐PCR‐based sequence, and identified 35 somatic mutations. Activating mutations in *RAS* family members and *GNAS* were observed in both PMCAs and DPAMs, and deregulation of TP53 or PI3K‐AKT pathway was found in PMCAs but not DPAMs. These data may be useful for the understanding of the development and progression of PMP.

## Materials and Methods

### Patients and clinical tissues

This study was approved by the institutional review boards of The Institute of Medical Science, The University of Tokyo (IMSUT‐IRB #26‐67), and National Center for Global Health and Medicine. All patients were given written informed consent for the study including genetic analysis. All samples were obtained from National Center for Global Health and Medicine (2012–2014). Tumor tissues and corresponding non‐cancerous tissues were obtained from surgical specimens of 18 patients with PMP, and frozen in deep freezer until analysis. In 12 of the 18 cases, primary tumors were available, but in the remaining six cases, metastatic lesions were used for the analysis. Clinicopathological information, treatment, and prognosis of the patients are summarized in Table 1. Eighteen patients underwent surgical operations including cytoreductive surgery (CRS) for 12 patients, debulking operation for 5, and exploratory laparotomy for one. Intraoperative hyperthermic intraperitoneal chemotherapy (HIPEC) with 10 mg/m^2^ mitomycin C at the temperature between 41 and 42°C was administered to the 12 CRS and 2 debulking operations. To seven patients treated with CRS and HIPEC, early postoperative intraperitoneal chemotherapy (EPIC) comprising 15 mg/kg fluorouracil was added intraperitoneally on days 1–4 after surgery. Residual disease was scored according to the completeness of cytoreduction (CCR) score 5. CCR0 indicates no macroscopic residual tumor; CCR1, no nodule larger than 2.5 mm in diameter remained; CCR2, nodules between 2.5 mm and 2.5 cm in diameter remained; and CCR3, nodules larger than 2.5 cm in diameter remained. All 18 tumors were histologically diagnosed as PMP and classified into 10 DPAMs and 8 PMCAs according to the definitions proposed by Misdraji *et al*. 3. Histological examination did not find component of signet ring cells in the 18 samples.

**Table 1:** Clinicopathological features of the 18 PMP patients

Patient ID	Age	Sex	Prior surgery	Treatment	CCR	Survival	Follow‐up (month)	Histological subtype
1	51	F	−	CRS + HIPEC	1	Alive (rec+)	37	DPAM
2	82	M	−	Debulking + HIPEC	2	Death	14	DPAM
3	74	F	−	CRS + HIPEC	0	Alive	25	DPAM
4	77	F	−	CRS + HIPEC	0	Alive	24	DPAM
5	57	M	−	Debulking	2	Death	18	DPAM
6	31	F	+	Exploratory laparotomy	2	Alive	1	DPAM
7	76	M	+	CRS + HIPEC	1	Alive	5	DPAM
8	67	M	+	CRS + HIPEC + EPIC	1	Alive	18	DPAM
9	72	M	−	Debulking + HIPEC	2	Alive	16	DPAM
10	70	M	−	CRS + HIPEC + EPIC	1	Alive	12	DPAM
11	71	M	−	CRS + HIPEC	1	Death	31	PMCA
12	70	F	−	CRS + HIPEC + EPIC	1	Alive (rec+)	12	PMCA
13	47	F	+	CRS + HIPEC + EPIC	1	Death	8	PMCA
14	76	F	−	Debulking	2	Alive	13	PMCA
15	65	M	+	Debulking	2	Alive	12	PMCA
16	55	M	−	CRS + HIPEC + EPIC	0	Alive	11	PMCA
17	60	F	−	CRS + HIPEC + EPIC	0	Alive	9	PMCA
18	61	F	−	CRS + HIPEC + EPIC	0	Alive	10	PMCA

CRS, cytoreductive surgery; HIPEC, hyperthermic intraperitoneal chemotherapy; EPIC, early postoperative intraperitoneal chemotherapy; CCR, completeness of cytoreduction; DPAM, disseminated peritoneal adenomucinosis; PMCA, peritoneal mucinous carcinomatosis; rec+, recurrence+.

### Extraction and quantification of DNA

Frozen sections (10 μm in thickness) of tumor and nontumorous tissues were fixed in ice‐cold 4% formalin for 10 min, washed by water for 5 min, and subsequently stained by hematoxylin. Tumor and non‐tumorous cells were collected from the sections by laser microdissection (LMD) using LMD 7000 (Leica Microsystems, Inc., Bensheim, Germany). Genomic DNA was extracted from the collected cells using a QIAamp DNA formalin‐fixed, paraffin‐embedded tissue kit (Qiagen, Valencia, CA) according to the manufacturer's instructions. Concentration of DNA was assessed by e‐SPECT (Malcom, Tokyo, Japan) and Qubit2 fluorometer (Invitrogen, CA). All genomic DNA was stored at −20°C until use.

### Multiplex PCR and DNA sequencing

Ten nanograms of DNA were used for multiplex PCR amplification of a panel covering 207 areas in 50 cancer‐related genes including *ABL1*,* AKT1*,* ALK*,* APC*,* ATM*,* BRAF*,* CDH1*,* CDKN2A*,* CBF1R*,* CTNNB1*,* EGFR*,* ERBB2*,* ERBB4*,* EZH2*,* FBXW7*,* FGFR1*,* FGFR2*,* FGFR3*,* FLT3*,* GNA11*,* GNAQ*,* GNAS*,* HNF1A*,* HRAS*,* IDH1*,* IDH2*,* JAK2*,* JAK3*,* KDR*,* KIT*,* KRAS*,* MET*,* MLH1*,* MPL*,* NOTCH1*,* NPM1*,* NRAS*,* PDGFRA*,* PIK3CA*,* PTEN*,* PTPN11*,* RB1*,* RET*,* SMAD4*,* SMARCB1*,* SMO*,* SRC*,* STK11*,* TP53*, and *VHL* (Ion AmpliSeq Cancer Panel v2; Life Technologies, Carlsbad, CA). The library construction and subsequent enrichment of the paired samples were performed using the OneTouch system according to the manufacturer's protocol. Sequencing was performed on their 316 chip with a capacity of 300‐500 megabases using the Ion PGM^™^ System (Life Technologies). Sequencing reads were mapped to the University of California, Santa Cruz (UCSC) human genome (GRCh37/hg19) using Torrent Suite^™^ software (Life Technologies).

### Variant calling and classification of somatic mutations

Sequence data were analyzed using Variant Caller^™^ and Ion Reporter^™^ (Life Technologies). Calls of single nucleotide variants (SNV) less than 2% and calls of insertions and deletions (indels) less than 5% in tumor tissues were excluded from further analysis. Calls at positions with sequence coverage greater than 50 reads in both tumor and non‐tumorous cells were analyzed, and those present in the tumor sample but not in the matched normal were regarded as somatic mutations. Fisher's exact test was carried out for the variants present in both tumor and corresponding normal tissue, and those detected at a significantly higher frequency in tumor tissues than the matched normal controls with p‐values less than 0.01 were classified as somatic mutations. All somatic mutations were reviewed by Integrative Genomic Viewer (IGV).

The somatic mutations were classified into three groups, namely intronic, splice site, and exonic mutations by their location, and the exonic mutations were further divided into four types, namely exonic indels, nonsense, synonymous, and non‐synonymous mutations. Pathological significance of the mutations was evaluated using several databases: ClinVar, COSMIC, ONCOMINE, Human Gene Mutation Database (HGMD), Leiden Open Variation Database (LOVD) in International Society for Gastrointestinal Hereditary Tumors (InSiGHT), TP53 Database in International Agency for Research on Cancer (IARC), dbSNP, and Human Genetic Variation Database (HGVD) in Kyoto University. Mutations reported to play a role in tumorigenesis, and those regarded deleterious in ClinVar, COSMIC, ONCOMINE, HGMD, LOVD, and TP53 Database were judged as pathological mutations. In addition, nonsense mutations, splice site mutations, and exonic indels were included as pathological mutations. Among the remaining mutations, we evaluated missense mutations using three prediction tools: SIFT, PolyPhen, and PANTHER. In this study, we considered those predicted to be damaged/deleterious/pathological by all three methods as pathological mutations, and other missense mutations as variants of uncertain significance (VUS). The remaining synonymous mutations were regarded as non‐pathological alterations.

### Histological analysis

Histological diagnosis was performed with all the tissue samples obtained by surgical resection and finally classified into PMCA and DPAM. Presence of signet ring cells was judged positive if the cells were observed at any percentages in the tumorous components but not in isolated mucin pools. Tissue sections of the 18 tumors were further analyzed by immunohistochemical staining with anti‐TP53 antibody (sc‐126, Santa Cruz Biotechnology, Inc., Delaware, CA), Histofine SAB‐PO(R) Kit (Nichirei, Tokyo, Japan), and ImmPACT DAB systems (VECTOR LABORATORIES, Burlingame, CA). Expression of TP53 was scored into three classes, namely normal, negative, and excessive, according to previous reports 6, 7. Normal staining referred to a weak focal nuclear staining, resembling a staining pattern for normal epithelium in less than 50% of tumor nuclei. A negative score indicated a complete lack of stained tumor nuclei (even though stromal cell nuclei showed normal pattern). The staining was classified as excessive if a strong nuclear staining was present in the majority of tumor cells (>50%).

### Statistical analysis

Statistical differences were analyzed using the Fisher's exact test. Differences were considered statistically significant when the p‐value was below 0.01.

## Results

### Treatments and clinicopathological features of the 18 PMP patients

**Figure 1:**
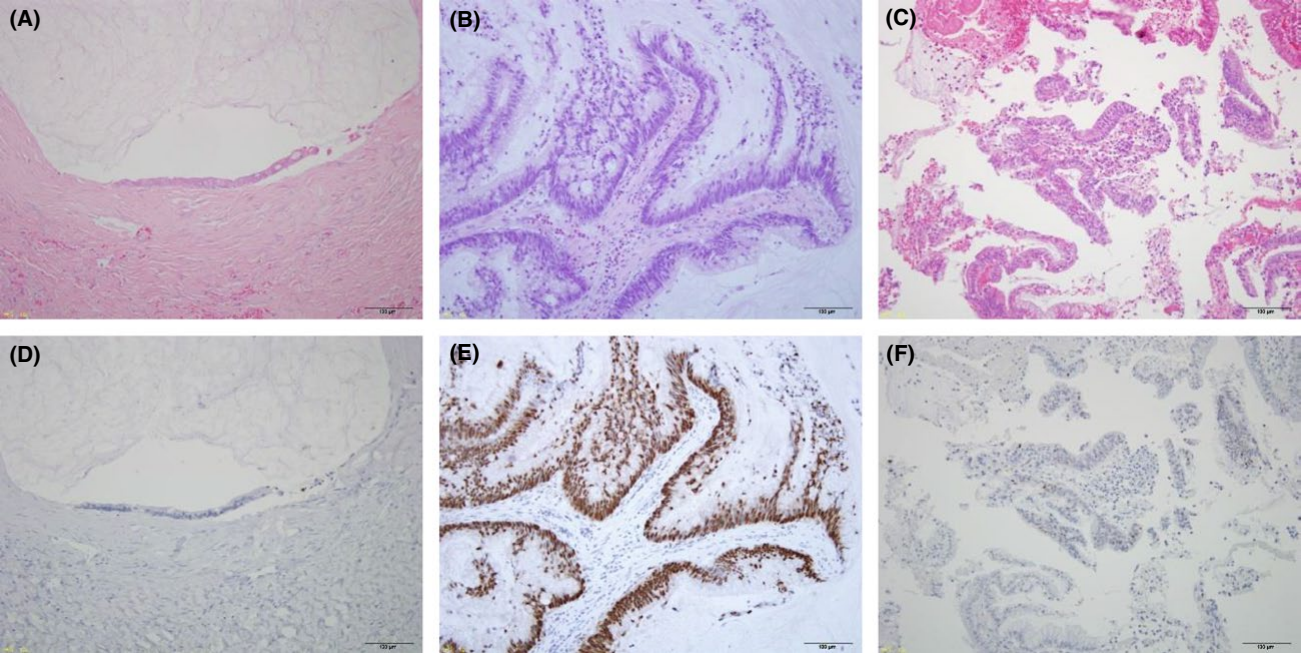
Hematoxylin and eosin (HE) staining (A–C) and immunohistochemical staining (D–F) of TP53. Typical histological appearance of DPAM (A) and PMCAs (B, C). Normal staining (D, F) and excessive staining (E). Magnification: ×200 (A–F).

The 18 PMPs originated from the appendix and were histologically classified into 10 DPAMs and 8 PMCAs. Components of signet ring cells were not detected in the 18 PMPs. A typical histological manifestation is shown in Figure 1. The gender and average age of the patients were not significantly different between the 10 DPAMs and the 8 PMCAs (Table 1). Patients underwent CRS (12 cases), debulking operation (5 cases), or exploratory laparotomy (1 case), and HIPEC was administered to the operations in 14 patients. EPIC was added to CRS‐HIPEC in seven cases. The follow‐up periods ranged from 1 to 37 months (median: 13 months). The periods are too short to compare the prognosis between the patients with DPAMs and those with PMCAs.

### Genetic analysis

Genetic analysis of the 18 tumors and the matched normal tissues was performed by amplicon sequencing with multiplex PCR using Cancer Panel v2. The average sequence throughput per sample was approximately 22 Mb, and the average number of reads per amplicon was 951. Among the 207 regions analyzed, 203 (98.3%) were covered by more than 100 reads on average, and all regions (207/207) were covered by at least 20 reads on average. Subsequent mutation analysis with Valiant Caller^™^ and Ion Reporter^™^ detected a total of 62 variant calls that were candidates for somatic mutations. However, 24 of the 62 calls were filtered out for further analysis because they were observed in matched normal tissues and the frequency was not statistically different between the tumors and non‐cancerous tissues. Among the remaining 38 alterations, three were located within homopolymers and they turned out to be miscalls by reviewing with IGV. We finally identified a total of 35 somatic mutations in the 18 tumors. All mutations are located in exons and comprised 33 non‐synonymous mutations, 1 deletion, and 1 insertion.

The most frequently mutated gene was *KRAS* (14/18) and the second was *GNAS* (8/18), followed by *TP53* (4/18), *SMAD4* (3/18), *AKT1* (1/18), *NRAS* (1/18), *PIK3CA* (1/18), *PDGFRA* (1/18), *RET* (1/18), and *VHL* (1/18) (Table 2). All 14 *KRAS* mutations were located at codons 12 or 13, the two major hot spots, and were missense mutations (p.G12D, p.G12V, p.G12S, and p.G13D). The eight *GNAS* mutations were located at codon 201 (p.R201H and p.R201C). Since these mutants are well‐known oncogenic forms, the mutations of *KRAS* and *GNAS* should play an active role in the tumorigenesis of PMP. All four *TP53* mutations (p.R175H, p.P250L, p.G266R, and p.R273C) were located in the DNA‐binding domain region and were reported to be pathological. Three *SMAD4* mutations included two indels and one missense mutation (p.C499R). This missense mutation was located in the MAD homology 2‐domain where many pathological mutations have been reported. We considered this mutation as a pathological mutation since all three prediction tools, PolyPhen SIFT, and PANTHER, judged it as a deleterious or pathological mutation. Thus, the three *SMAD4* mutations should result in loss of SMAD4 function and play a vital role in the tumorigenesis.

**Table 2:** List of pathological mutations

Gene	Nucleotide	Amino acid	Type of mutation	Evidence^a^	No. of cases
*KRAS*	c.34G>A	p.G12S	Missense	ONC	1
	c.35G>A	p.G12D	Missense	ONC	6
	c.35G>T	p.G12V	Missense	ONC	6
	c.38G>A	p.G13D	Missense	ONC	1
*GNAS*	c.601C>T	p.R201C	Missense	ONC	2
	c.602G>A	p.R201H	Missense	ONC	6
*TP53*	c.524G>A	p.R175H	Missense	ONC, IARC	1
	c.749C>T	p.P250L	Missense	ONC, IARC	1
	c.796G>C	p.G266R	Missense	ONC, IARC	1
	c.817C>T	p.R273C	Missense	ONC, IARC	1
*SMAD4*	c.553_556delCCAC	p.P185QfsX16	Deletion	INDEL	1
	c.1586_1587insA	p.L529fs	Insertion	INDEL	1
	c.1495T>C	p.C499R	Missense	CSMC, IS	1
*AKT1*	c.49G>A	p.E17K	Missense	ONC	1
*NRAS*	c.181C>A	p.Q61K	Missense	ONC	1
*PDGFRA*	c.1658C>T	p.P553L	Missense	IS	1
*PIK3CA*	c.3127A>G	p.M1043V	Missense	CSMC, IS	1
*RET*	c.2651A>T	p.E884V	Missense	CSMC, IS	1
*VHL*	c.430G>A	p.G144R	Missense	ONC	1

a Evidence of pathological significance was judged from the type of mutation (INDEL), databases including ONCOMINE (ONC), TP53 database in International Agency for Research on Cancer (IARC), and COSMIC (CSMC), or *in silico* (IS) analyses.

We identified in this study a missense *PDGFRA* mutation of c.1658C>T (p.P553L) that has not been reported in literatures or public databases. *In silico* analysis of the mutation using the three algorithms predicted it as a pathological mutation; probably damaging (score:1) by PolyPhen, deleterious (score:0) by SIFT, and deleterious (subPSEC: −6.28, P_deterious_: 0.96) by PANTHER. Taken together, all 35 somatic mutations were classified as pathological mutations.

### Mutation profiles of DPAMs and PMCAs

We further compared mutation profiles between the 10 DPAMs and the 8 PMCAs. It is of note that *KRAS* mutations were observed in both types of tumors (eight of the ten DPAMs and six of the eight PMCAs), and that a mutation of *NRAS* was found in a PMCA without *KRAS* mutation. These data suggest that the activation of RAS family members is commonly involved in both DPAMs and PMCAs. *GNAS* mutations were detected in five DPAMs and three PMCAs, implying that activation of GNAS may also play a key role in the development of both types of tumors. On the other hand, *TP53* mutations were found in three PMCAs but not in the 10 DPAMs, suggesting that the inactivation of TP53 may render malignant properties to PMCAs. Similarly, mutations in *PIK3CA, AKT1,* or *PDGFRA* were found in the PMCAs, but not in the DPAMs. It is worthwhile to note that mutations of *PIK3CA* or *AKT1* were found in two PMCAs without *TP53* mutations but not in the DPAMs, and that the mutations were mutually exclusive. Therefore, activation of the PI3K‐AKT pathway may also play an important role in the malignant transformation of PMP.

### Accumulation of TP53 in PMCAs

To corroborate the deregulation of TP53 in PMCAs, we performed immunohistochemical staining of TP53 using paraffin‐embedded tissues (Fig. 1). Consistent with the mutation data, strong accumulation of TP53 was not observed in the DPAMs, but three of the eight PMCAs revealed excessive TP53 staining. As expected, we found the accumulated TP53 protein in the three tumors carrying *TP53* mutation (Table 3).

**Table 3:** Mutation status and IHC of TP53

Histological classificationPatient ID		DPAM										PMCA							
		1	2	3	4	5	6	7	8	9	10	11	12	13	14	15	16	17	18
Mutation status	*KRAS*	Mut	Mut	Mut	Mut		Mut	Mut	Mut	Mut			Mut	Mut	Mut	Mut	Mut	Mut	
	*GNAS*			Mut				Mut	Mut	Mut	Mut				Mut	Mut	Mut		
	*TP53*												Mut					Mut	Mut
	*PIK3CA*													Mut					
	*AKT1*											Mut							
	*SMAD4*	Mut								Mut					Mut				
	*NRAS*																		Mut
	*PDGFRA*															Mut			
	*RET*		Mut																
	*VHL*	Mut																	
TP53 IHC		N	N	N	N	N	N	N	N	N	N	N	E	N	N	N	N	E	E

Mut, a pathological mutation (somatic) was identified; blank, no somatic mutation was identified; N, normal staining; E, excessive staining.

## Discussion

In this study, we performed genetic analysis of 18 Japanese PMPs and unveiled their mutation profiles. Analysis of the data disclosed that both DPAMs and PMCAs carry frequent mutations in *RAS* family members and *GNAS*, and that mutations in genes associated with TP53 and/or PI3K‐AKT pathways may play a crucial role in the malignant transformation of PMP.

In accordance with our data, several groups have already reported frequent *KRAS* mutations in PMP at a frequency between 58% and 94% (37 [57.8%] of 64 PMP, Shetty *et al*. 8; 33 [94.3%] of 35 PMP, Nishikawa *et al*. 9; 70%, Sio *et al*. 10; and 75%, Liu *et al*. 11). Since the frequency of *KRAS* mutation in colorectal cancer is approximately 40%, *KRAS* mutation in PMP is markedly high. In regard to the difference of *KRAS* mutation between PMCAs and DPAMs, two groups showed more frequent mutations in PMCAs than DPAMs, but other groups did not observe significant difference between the two types of tumors 8, 9. Our data support the latter view and revealed frequent mutations in DPAMs as well as PMCAs. Although we did not find *KRAS* mutations in four tumors, one of the four had an *NRAS* mutation, suggesting that the remaining three tumors may carry mutations in other regions of *KRAS* or *NRAS*, or in other genes associated with the RAS‐MAPK pathway. These data imply that the activation of RAS‐MAPK pathway plays a crucial role in both types of PMP, and that anticancer drugs targeting this pathway should be a rational strategy to treat PMP.

In good agreement with previous reports 9, 10, 11, we identified *GNAS* mutations at a similar frequency in PMPs (44.4%). Although Nishikawa *et al*. 9 identified *GNAS* mutations in DPAMs but not in PMCAs, we found *GNAS* mutations in both PMCAs (3/8, 37.5%) and DPAMs (5/10, 50%). This result suggested that *GNAS* mutation may also play an important role in the development of both types of PMP. In this study, *GNAS* mutations were not associated with any clinicopathological factors including gender, age, and histological stages (data not shown). The *GNAS* gene encodes the α‐subunit of stimulatory G protein, and the R201C and R201H mutants at codon 201, the mutation hot spot, enhance the activity of adenylyl cyclase. Consequently, mutated GNAS leads to the activation of the protein kinase A (PKA) pathway through the induction of cyclin AMP (cAMP). Recently, a group reported that GNAS regulates expression of *MUC2* and *MUC5AC* in colon cancer cells, and that its expression does not associate with cell proliferation both *in vitro* and *in vivo* 9. *GNAS* mutations are also frequently identified in intraductal papillary mucinous neoplasms (IPMN) of the pancreas 12. Since it is reported that the secreted mucin from PMP contains MUC2, MUC5AC, and MUC5B, activated GNAS may induce the production of these mucin in both PMP and IPMN.

In this study, *TP53* mutations were shown in three of the eight PMCAs, but not in any of the ten DPAMs. Consistently, immunohistochemical staining showed excessive accumulation of TP53 protein in the three PMCAs, but not in other PMCAs with wild‐type *TP53* or the 10 DPAMs, corroborating that impaired TP53 pathway is associated with PMCA. Two groups have reported low frequencies of *TP53* mutations in PMP so far; one of ten high‐grade PMPs 7, and two of eight low‐grade/well‐differentiated mucinous adenocarcinoma with PMP 11. On the other hand, several groups found accumulation of TP53 at a higher frequency in PMCA than DPAM by immunohistochemical staining 7, 8. Although the frequency of TP53 accumulation is different among the studies, possibly because of different antibodies and/or condition of the tissues analyzed, these studies have suggested that TP53 deregulation is more frequently associated with PMCA than DPAM. Since *TP53* mutation is involved in the step of malignant transformation, impaired TP53 pathway may render malignant properties to PMCA. In addition, immunohistochemical staining of TP53 may be useful as a biomarker of PMCA.

Although the number of mutations is small, we identified mutations in *PIK3CA* and *AKT1* in PMCAs, but not in DPAMs. PI3K‐AKT pathway plays a crucial role in regulating a number of cellular processes, such as apoptosis, migration, angiogenesis, cell proliferation, and glucose metabolism. It is of note that 14–32% of colorectal cancer harbored somatic mutations in *PIK3CA* and that the mutations are rare in premalignant lesions of the colon 13, 14. Thus, deregulation of PI3K‐AKT pathway may also play a crucial role in the progression of PMP. Since the oncogenic mutations in *PIK3CA* induce downstream signaling through the phosphorylation of AKT and subsequent activation of mTOR complex 1 (mTORC1), inhibition of PI3K, AKT, and/or mTORC1 may be an effective therapeutic strategy to treat the tumors with an oncogenic *PIK3CA* mutation. Additionally, inhibitors of AKT and/or mTORC1 may benefit the patients with an oncogenic *AKT1* mutation.

In this study, we identified a novel mutation in *PDGFRA* of c.1658C>T (p.P553L). Although the most frequent mutation of *PDGFRA* (p.D842V) is located in the tyrosine kinase 2 domain, the juxtamembrane domain (exon12) also harbors frequent mutations such as p.D561V. Taking the location together with the *in silico* data using the three tools into our consideration, we judged that p.P553L mutation was a pathological mutation. *PDGFRA* mutations are reported in human cancers including colorectal cancer (6.0%) and stomach cancer (2.6%) in the public data of COSMIC v71. It is of note that frequent *PDGFRA* mutations have been reported in gastrointestinal stromal tumors (GIST) (10%) 15. Most of the mutations are considered to activate its kinase activity and autonomously induce its downstream molecules. Importantly, tumors carrying *PDGFRA* mutations were shown to be sensitive to the *in vitro* treatment with Imatinib, an inhibitor of kinase activity of bcr‐abl, cKit, and PDGFRA 16, 17. Therefore, patients with the c.1658C>T (p.P553L) mutation may benefit from Imatinib treatment.

Recently, PMPs with signet ring cells are reported to be associated with aggressive characteristics and poor outcomes by Davison *et al*. and Shetty *et al*. 18, 19. Although we did not find presence of signet ring cells in the 18 PMPs, further studies are necessary for the better classification of PMP and the elucidation of biological properties and genetic profiles of PMP with signet cell component.

In conclusion, we have found in the present study that *KRAS* and/or *GNAS* mutations are frequently involved in the development of PMPs, and that disruption of TP53 or PI3K‐AKT pathway may play a crucial role in their progression. In addition, we have shown here that immunohistological staining of TP53 may be useful for the classification of PMPs. Importantly, analysis of mutation profiles may be useful not only for the comprehensive understanding of their tumorigenesis, but also the personalized therapy for patients with PMP.

## Conflict of Interest

None declared.
